# MiR-19a regulates PTEN expression to mediate glycogen synthesis in hepatocytes

**DOI:** 10.1038/srep11602

**Published:** 2015-06-26

**Authors:** Lin Dou, Xiangyu Meng, Xiaofang Sui, Shuyue Wang, Tao Shen, Xiuqing Huang, Jun Guo, Weiwei Fang, Yong Man, Jianzhong Xi, Jian Li

**Affiliations:** 1 Department of Biomedical Engineering, College of Engineering, Peking University, Beijing, China; 2 Peking University Fifth School of Clinical Medicine, Beijing, China; 3 Key Laboratory of Geriatrics, Beijing Institute of Geriatrics & Beijing Hospital, Ministry of Health, Beijing, China; 4 First Affiliated Hospital of Jiamusi University, Jiamusi, China

## Abstract

MiR-19a, a member of mir-17-92 microRNA clusters, has been demonstrated to promote cell proliferation and angiogenesis via regulating the PI3K/AKT pathway, the major insulin signaling pathway. However, whether miR-19a plays an important role in glycogen synthesis in hepatocytes remains unknown. Here, we define the impact of miR-19a on glycogen synthesis and IL-6-induced reduced glycogenesis in hepatocytes and its underlying mechanisms. Our studies indicate that miR-19a was down-regulated in the livers of db/db mice and mice injected with IL-6, as well as mouse NCTC 1469 hepatocytes and HEP 1–6 hepatocytes treated by IL-6. We found that over-expression of miR-19a in NCTC 1469 cells and HEP 1–6 cells led to increased activation of the AKT/GSK pathway and synthesis of glycogen, whereas down-regulation of miR-19a impaired AKT/GSK phosphorylation and glycogenesis. Over-expression of miR-19a ameliorated IL-6-induced reduced glycogen synthesis in hepatocytes. Moreover, we identified PTEN as the target of miR-19a by a luciferase assay. Down-regulation of PTEN rescued the effects of miR-19a suppression on the activation of the AKT/GSK pathway and improved glycogenesis in NTC 1469 cells. These findings show for the first time that miR-19a might activate the AKT/GSK pathway and glycogenesis via down-regulation of PTEN expression.

The liver is a vital organ that plays a central role in maintaining lipid and glucose metabolism. Glycogenesis is one of the major functions of the liver. Impaired glycogen levels are the hallmark of insulin resistance in hepatocytes. Insulin resistance leads to metabolic abnormalities, such as hyperglycemia, hyperinsulinemia, and hypertriglyceridemia which are common features of type 2 diabetes and metabolic syndrome^1^.

IL-6 has been implicated in the pathogenesis of insulin resistance *in vitro* and *in vivo*^2^,^3^. Elevated plasma IL-6 levels play an important role in insulin resistance by impairing insulin signaling^4^. Moreover, our previous study indicated that IL-6 induced insulin resistance in cultured human HepG2 hepatocytes, mouse NCTC 1469 hepatocytes and primary hepatocytes, as assessed by their decreased capacity to accumulate glycogen in the presence of insulin^5^.

MicroRNAs (miRNAs) negatively regulate gene expression at the post-transcriptional level, either by inhibiting translation or by degrading the target mRNA^6^. There is increasing evidence that microRNAs are involved in the progression of insulin resistance. For instance, miR-320 regulates insulin resistance in adipocytes through targeting of a kinase subunit of PI3K, p85^7^. In addition, decreased levels of miR-103/107 in adipocytes increased cavolin-1 expression, which can stabilize the insulin receptor and enhance insulin signaling^8^. Our previous study indicated that miR-200s contribute to hepatic insulin resistance induced by IL-6 via targeting FOG2^5^. It has been considered that miR-19a is a member of the miR-17-92 cluster, which is located on chromosome 13q31.3 and is involved in the pathogenesis of tumors. Several studies have shown that miR-19a and miR-19b were up-regulated in different types of tumors^9^,^10^. Moreover, miR-19a promotes cell proliferation and angiogenesis via regulating the PI3K/AKT pathway, the major insulin signaling pathway^11^. However, whether miR-19a plays an important role in glycogen synthesis in hepatocytes remains unknown.

Here, we define the impacts of miR-19a on glycogen synthesis and IL-6-induced reduced glycogenesis in hepatocytes and its underlying mechanisms. Our findings suggest, for the first time to our knowledge, that miR-19a plays an important role in glycogenesis via down-regulation of PTEN expression.

## Results

### MiR-19a is down-regulated in the livers of db/db mice and mice injected with IL-6 as well as mouse NCTC 1469 hepatocytes and HEP 1–6 hepatocytes treated with IL-6

**Figure 1:**
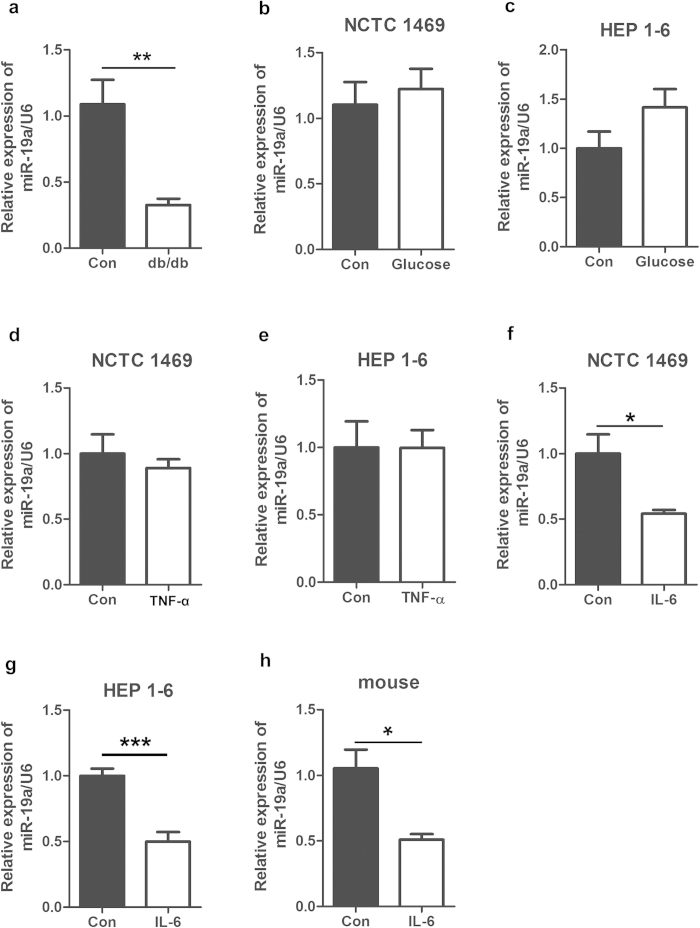
MiR-19a is down-regulated in the livers of db/db mice and mice injected with IL-6 as well as mouse NCTC 1469 hepatocytes and HEP 1-6 hepatocytes treated with IL-6. (**a**) Reduced miR-19a expression in the livers of db/db mice, as verified by real-time PCR. (**b**–**g**) MiR-19a expression in the mouse NCTC 1469 hepatocytes and HEP 1–6 hepatocytes treated with 33.3 mM glucose for 48 h (**b** and **c**), 10 ng/ml hIL-6 for 24 h (**d** and **e**) and 10 ng/ml TNF-α for 24 h (**f** and **g**). (**h**) Expression of miR-19a in the livers of 12-week-old male C57BL/6J mice injected with 16 μg/ml IL-6 by pumps for 7 days. Data represent the mean ± S.D. N = 3 independent experiments or N = 5 mice.*p < 0.05; **p < 0.01 by ANOVAtest (vs. control).

To profile the changes of the microRNA expression in hepatic insulin resistance, miRNA microarrays were used to analyze miRNAs in the livers of db/db mice (n = 5) and control mice (n = 5). Supplemental Table1 shows that the level of miR-19a was decreased in the livers of db/db mice. Consistently, real-time PCR verified reduced miR-19a expression in the livers of db/db mice (Fig. 1a). Several factors can lead to insulin resistance, such as high glucose and inflammatory factors, such as TNF-α and IL-6. According to our previous study, decreased glycogenesis and impaired activation of the PI3K/AKT pathway in liver cells can be induced by glucose, TNF-α and IL-6 (Supplemental Fig S1). Therefore, mouse NCTC 1469 cells and Hep 1–6 cells were treated with 33.3 mM glucose for 48 h, 10 ng/ml hIL-6 for 24 h and 10 ng/ml TNF-α for 24 h to induce insulin resistance. As shown in Fig. 1b–g, treatment with IL-6 but not glucose and TNF-α led to down-regulated miR-19a expression. To confirm the effect of IL-6 on the expression of miR-19a *in vivo*, 12-week-old male C57BL/6J mice were injected with 16 μg/ml IL-6 by pumps for 7 days. Expression of miR-19a was also reduced in the livers of mice injected with IL-6 (Fig. 1h). Our data demonstrate the possibility that down-regulation of miR-19a is involved in the pathogenesis of hepatic insulin resistance.

### MiR-19a mediates activation of the AKT/GSK pathway and synthesis of glycogen in hepatocytes

**Figure 2:**
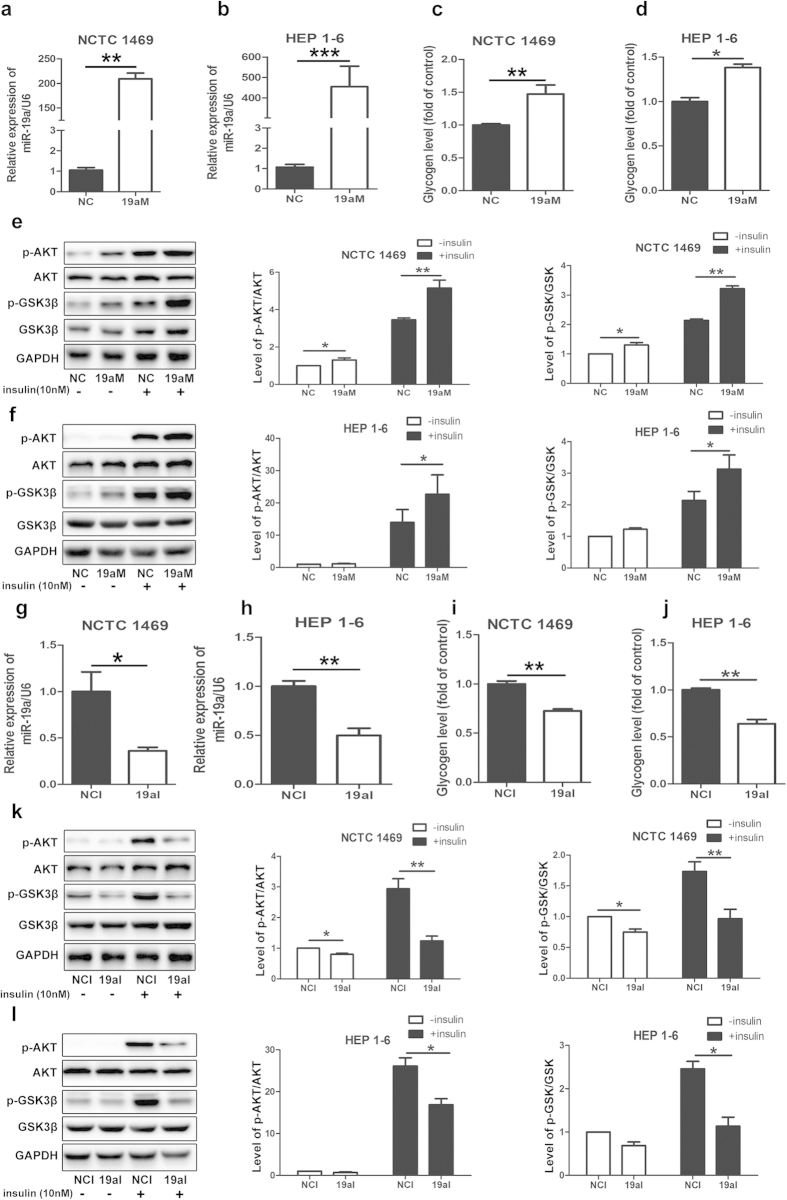
MiR-19a mediates activation of the AKT/GSK pathway and synthesis of glycogen in hepatocytes. (**a** and **g**) Levels of miR-19a in mouse NCTC 1469 cells and HEP 1–6 cells transfected with miR-19a mimics or miRNA mimic control. Activation of the AKT/GSK pathway (**b** and **h**) and synthesis of glycogen (**e** and **k**) in the NCTC 1469 cells and HEP 1–6 cells transfected with miR-19a mimics. (**c** and **i**) Expression of miR-19a in the NCTC 1469 cells transfected with miR-19a inhibitor or miRNA inhibitor control. Phosphorylation of the AKT/GSK pathway (**d** and **j**) and synthesis of glycogen (**f** and **l**) in the NCTC 1469 cells and HEP 1–6 cells transfected with miR-19a inhibitor. Data represent the mean ± S.D. N = 3 independent experiments. *p < 0.05; **p < 0.01byANOVAtest (vs. control). Full-length blots are presented in the supplementary information (supplementary Figure S2).

Next, we investigated the effects of miR-19a on the activation of the PI3K/AKT pathway and glycogenesis in NCTC 1469 cells and HEP 1–6 cells. MiR-19a mimics and inhibitor were transfected into both types of cells for 48 h. As shown in Fig. 2a and 2b, the level of miR-19a was increased to more than 100-fold in the two cell lines transfected with miR-19a mimics compared with those transfected with miRNA mimic control. In parallel with the increased miR-19a expression, the synthesis of glycogen and the activation of the AKT/glycogen synthase kinase (GSK) pathway were elevated in both NCTC 1469 cells and HEP 1–6 cells transfected with miR-19a mimics (Fig. 2c–f). In contrast, the level of miR-19a was decreased to 30-40% compared with those transfected with miRNA inhibitor control (Fig. 2g,h). Inhibition of miR-19a blocked glycogenesis (Fig. 2i,j) and the activation of the PI3K/AKT pathway (Fig. 2k,l).

### MiR-19a ameliorates IL-6-induced impaired phosphorylationof the AKT/GSK pathway and synthesis of glycogen in hepatocytes

**Figure 3:**
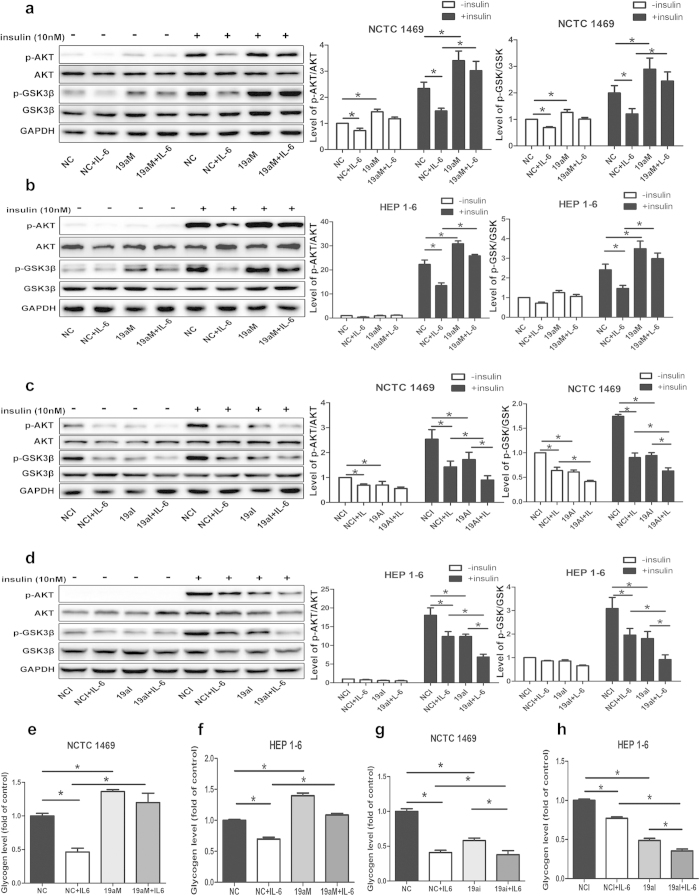
MiR-19a ameliorates IL-6-induced impaired phosphorylation of the AKT/GSK pathway and synthesis of glycogen in hepatocytes. Activation of the AKT/GSK pathway (**a** and **b**) and synthesis of glycogen (**e** and **f**) in the NCTC 1469 cells and HEP 1–6 cells treated with 10 ng/ml IL-6 for 24 h followed by transfection with miR-19a mimics for 48 h. AKT/GSK pathway activation (**c** and **d**) and glycogenesis (**g** and **h**) in the NCTC 1469 cells and HEP 1–6 cells treated with 10 ng/ml IL-6 for 24 h followed by transfection with miR-19a inhibitor for 48 h. Data represent the mean ± S.D. N = 3 independent experiments. *p < 0.05; **p < 0.01by ANOVAtest (vs.IL-6). Full-length blots are presented in the supplementary information (supplementary Figure S3).

To further determine the role of miR-19a in IL-6-induced insulin resistance, NCTC 1469 cells and HEP 1–6 cells were treated with 10 ng/ml IL-6 for 24 h followed by transfection with miR-19a mimics or inhibitor for 48 h. Over-expression of miR-19a ameliorated IL-6-induced impaired activation of the AKT/GSK pathway (Fig. 3a,b) and synthesis of glycogen (Fig. 3e,f) in NCTC 1469 cells and HEP 1–6 cells. However, down-regulation of miR-19a further promoted IL-6-induced reduced AKT/GSK pathway activation (Fig. 3c,d) and glycogenesis (Fig. 3g,h) in NCTC 1469 cells and HEP 1–6 cells.

### PTEN is identified as a target gene of miR-19a

**Figure 4:**
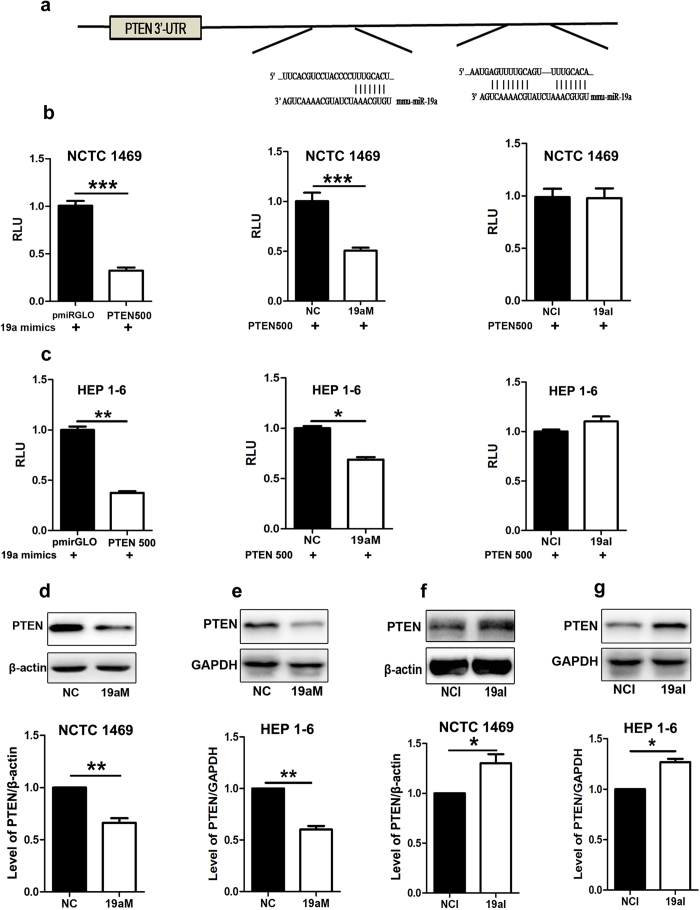
PTEN is identified as a target gene of miR-19a. (**a**) Several binding sites of miR-19a on 3’-UTR of PTEN, as analyzed by miRNA target prediction databases, including Miranda, TargetScan and PicTar. The fragment of PTEN 3’-UTR from 500 nt to 1600 nt was cloned and inserted into pmiRGLO vector (PTEN500). A luciferase reporter assay was used to assess whether miR-19a can directly bind to the 3’-UTR of PTEN. (**b** and **c**) MiR-19a mimics significantly inhibited the luciferase activity in the NCTC1469 cells and HEP 1–6 cells transfected with PTEN500 vector compared with pmiRGLO (left). Transfection with miR-19a mimics dramatically reduced the luciferase activity in the NCTC1469 cells and HEP 1–6 cells transfected with the luciferase reporter vector containing the 3’-UTR of PTEN compared with those transfected with miRNA mimic control (middle). MiR-19a inhibitor did not affect luciferase activity (right). (**d** and **e**) Decreased level of PTEN in the NCTC1469 cells and HEP 1–6 cells transfected with miR-19a mimics. (**f** and **g**) Increased level of PTEN in the NCTC1469 cells and HEP 1–6 cells transfected with miR-19a inhibitor. Data represent the mean ± S.D. N = 3 independent experiments. *p < 0.05; **p < 0.01; ***p < 0.001by ANOVAtest (vs. control). Full-length blots are presented in the supplementary information (supplementary Figure S4).

Three binding sites of miR-19a on the 3'-UTR of PTEN were analyzed by miRNA target prediction databases, including Miranda, TargetScan and PicTar (Fig. 4a). The fragment of PTEN 3'-UTR from 500 nt to 1600 nt was cloned and inserted into pmiRGLO vector (PTEN500). A luciferase reporter assay was used to assess whether miR-19a can directly bind to the 3'-UTR of PTEN. As shown in Fig. 4b,c, over-expression of miR-19a dramatically reduced the luciferase activity in the NCTC1469 cells and HEP 1–6 cells transfected with the luciferase reporter vector containing the 3'-UTR of PTEN. Moreover, the level of PTEN was decreased in the NCTC1469 cells and HEP 1–6 cells transfected with miR-19a mimics (Fig. 4d,e), whereas down-regulation of miR-19a elevated the level of PTEN (Fig. 4f,g), suggesting that miR-19a negatively regulates the expression of PTEN by directly binding to its 3’-UTR.

### PTEN participates in miR-19a-mediated glycogenesis in hepatocytes

**Figure 5:**
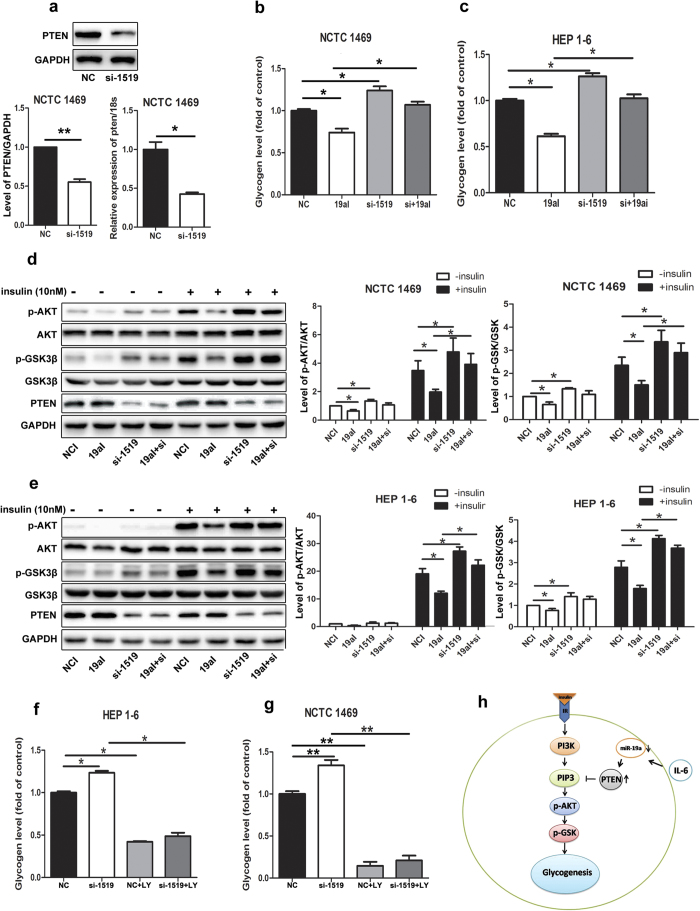
PTEN participates in miR-19a-mediated glycogenesis in hepatocytes. Protein levels and mRNA levels (**a**) of PTEN in the NCTC 1469 cells transfected with siRNA (si-1519) targeting PTEN mRNA or with negative siRNA control. Down-regulation of PTEN rescued the effects of miR-19a inhibitor suppression on the activation of the AKT/GSK pathway (**d** and **e**) and improved glycogenesis (**b** and **c**) in NCTC 1469 cells and HEP 1–6cells. PTEN did not affect the glycogenesis after treatment with PI3K inhibitor LY294002 (**f** and **g**) . The molecular mechanism by which PTEN participates in miR-19a-mediated glycogenesis in hepatocytes (**h**). Data represent the mean ± S.D. N = 3 independent experiments. *p < 0.05; **p < 0.01by ANOVAtest (vs. control or miR-19a inhibitor or LY294002). Full-length blots are presented in the supplementary information (supplementary Figure S5).

To gain further insights into the role of PTEN in miR-19a-mediated glycogenesis, siRNA (si-1519) targeting PTEN mRNA was transfected into NCTC 1469 cells and HEP 1–6 cells. Both the protein levels and mRNA levels of PTEN were decreased by 30–40% compared with those transfected with negative siRNA control (Fig. 5a). Down-regulation of PTEN rescued the effects of miR-19a suppression on the activation of the AKT/GSK pathway and improved glycogenesis in NCTC 1469 cells and HEP 1–6 cells (Fig. 5d,e). Moreover, PTEN did not affect glycogenesis after treatment with PI3K inhibitor LY294002 (Fig. 5f,g). In conclusion, as shown in Fig. 5h, PTEN participates in miR-19a-mediated glycogenesis in hepatocytes via regulation of the AKT/GSK pathway.

## Discussion

Insulin resistance may occur in certain tissues, such as fat, muscle, and liver. Insulin resistance in the liver leads to impaired glycogen synthesis and failure to suppress glucose production^12^. In the liver, insulin is an activator of the PI3K/AKT signaling cascade that regulates glucose metabolism, glycogen/lipid and protein synthesis and gene activation required for growth and differentiation^13^. There is increasing evidence that miRNAs, including miR-320, miR-200s and miR-103/107, are involved in the progression of insulin resistance^6^,^7^,^8^. To preliminarily profile the expression of miRNAs in the livers of db/db mice and control mice, the liver samples from five db/db mice and five control mice were pooled together by equal amount, respectively. These two mixed liver samples were analyzed by the miRCURY^TM^ LNA Array. Therefore, we cannot perform statistical analyses for the data of the microarray analysis. This is the limitation in our microarray analysis. However, to confirm the results of microarray analysis, we used real-time PCR to measure the expression levels of several screened miRNAs in every liver sample from five db/db mice and five control mice. Based on the preliminary data of the microarray analysis and miRNA target prediction, we screened several miRNAs down-regulated in the livers of db/db mice, and targeted PTEN. These miRNAs were further measured by real-time PCR. The results showed that some of screened miRNAs did not change in the livers of db/db mice compared with the livers of control mice, for example miR-106b and miR-181a (data not shown). Moreover, in the previous study, we transfected mimics and inhibitors of screened miRNAs into NCTC 1469 cells and HEP 1–6 cells to determine their effects on the activation of insulin signal pathway. The results indicated that miR-19a was one of the miRNAs which significantly affected the activation of the PI3K/AKT pathway. Therefore, in the present study, we further investigated the contribution of miR-19a to insulin resistance via direct regulation of PTEN expression. Otherwise, we also explored the role of other microRNAs such as miR-200s in hepatic insulin resistance. In a previous study, we found that db/db mice exhibited decreased levels of miR-200s, including miR-200a, miR-200b and miR-200c, impaired activation of the AKT/GSK pathway and reduced glycogenesis in the livers, accompanied by elevated serum concentrations of IL-6^8^. The present study also showed reduced expression of miR-19a in the livers of db/db mice. Increasing evidence indicates that serum levels of IL-6 are associated with insulin resistance and obese type 2 diabetes^2^,^14^. IL-6 alters insulin sensitivity in hepatocytes by impairing the PI3K/AKT signaling through serine phosphorylation of IRS-1 and activation of SOCS proteins^1^,^15^. We found that treatment with IL-6 impaired the activation of the PI3K/AKT pathway and decreased the levels of glycogen *in vitro* and *in vivo*^8^. Because db/db mice are complex and accompanied by other factors, such as elevated levels of serum glucose and inflammatory factor TNF-α, it is difficult to determine the involvement of IL-6 in the down-regulation of miR-19a. Therefore, we extended these observations from db/db mice to two hepatocyte cell lines, NCTC 1469 and HEP 1–6. The results show that the treatment with IL-6 but not glucose and TNF-α led to down-regulated miR-19a expression in liver cells. The down-regulation of miR-19a by IL-6 was assessed in the livers of C57BL/6J mice injected with IL-6. These results suggest that IL-6 suppresses the expression of miR-19a *in vivo* and *in vitro*.

MiR-19a, a member of mir-17-92 microRNA clusters, is considered as oncogenic miRNA, which promotes cell proliferation and angiogenesis^16^. A previous study found that miR-19a and miR-19b participated in glioma genesis via negative regulation of PTEN^17^. Jian He et al reported that shear stress increased the expression of miR-19a and improved the PI3K pathway. Moreover, inhibition of the PI3K pathway attenuated the shear-induced miR-19a^18^. Our studies indicate that over-expression of miR-19a in NCTC 1469 cells and HEP 1–6 cells led to increased activation of the AKT/GSK pathway and synthesis of glycogen, whereas down-regulation of miR-19a impaired AKT/GSK phosphorylation and glycogenesis. More importantly, over-expression of miR-19a ameliorated IL-6-induced reduced glycogen synthesis in hepatocytes.

What is the target gene that is involved in the role of miR-19a in glycogenesis? MiRNA target prediction databases, including Miranda, TargetScan and PicTar, were used to analyze miR-19a target genes. The analysis predicted PTEN as the target of miR-19a, and there are several binding sites for the miR-19a at the PTEN 3'-UTR. PTEN has been considered as a tumor suppressor and one of the most frequently mutated genes in tumors^19^,^20^. PTEN is expressed in all tissues in the body and contains a tensin-like domain and a phosphatase catalytic domain^21^. PTEN antagonizes the insulin-activated PI3K/AKT pathway by catalyzing PIP3 dephosphorylation and converting it into PIP2^22^. Guenzl et al reported that insulin hyper-sensitivity in mice was induced by the cell type-specific deletion of PTEN in hepatic tissue^23^. Our results suggest that miR-19a negatively regulates the expression of PTEN by directly binding to its 3'-UTR. The expression of miR-19a was increased more than 100 times by transfection with miR-19a mimics. MiR-19a mimics have a significant effect on luciferase activity. However, transfection with miR-19a inhibitor only decreased the expression of miR-19a to 30–40%. In addition, miR-19a has three binding sites in the PTEN 3’-UTR, but our luciferase vector only contains two binding sites. Therefore, miR-19a inhibitor did not significantly affect luciferase activity. However, miR-19a inhibitor increases the PTEN protein level significantly. To gain further insights into the role of PTEN in miR-19a-mediated glycogenesis, siRNA (si-1519) targeting PTEN mRNA was transfected into NCTC 1469 cells and HEP 1–6 cells. Down-regulation of PTEN rescued the effects of miR-19a suppression on the activation of the AKT/GSK pathway and improved glycogenesis in NCTC 1469 cells and HEP 1–6 cells. Moreover, PTEN did not affect glycogenesis after treatment with PI3K inhibitor LY294002. In conclusion, as shown in Fig. 5h, PTEN participates in miR-19a-mediated glycogenesis in hepatocytes via regulation of the AKT/GSK pathway. These findings provide mechanistic insight into the effects of miR-19a on the regulation of the AKT/GSK pathway and the synthesis of glycogen in hepatocytes. MiR-19a might activate the AKT/GSK pathway and glycogenesis via down-regulating PTEN expression. However, we need to further investigate the effects of miR-19a over-expression and PTEN knockdown in the livers of db/db mice or PTEN over-expression in the normal mice on glucose metabolism.

## Methods

### Animals

Eighteen-week-old db/db mice (C57BL/KsJ) were obtained from the Peking University Health Science Center (originally purchased from Jackson Laboratory). Briefly, db/db mice (n = 5) and age-matched wild-type (WT) mice (n = 5) were fed a standard laboratory diet for 18 weeks.

Twelve-week-old male C57BL/6J mice were provided by Peking University Health Science Center. The mice (n = 10) were separated into two groups and fed a standard laboratory diet in a temperature-controlled (20–24 °C) and humidity-controlled (45–55%) environment. A 12 h/12 h light/dark cycle was maintained. For the experiment examining chronic IL-6 exposure, Alzet osmotic pumps (Durect, Cupertino, CA) with a 7-day pumping capacity and infusion rate of 1 μl/h were used. Pumps were filled to capacity with 16 μg/ml hIL-6 diluted in carrier (0.9% NaCl and 0.1% BSA)^24^. Following induction of halothane general anesthesia, pumps were implanted into the intrascapular subcutaneous space. Incisions were closed with interrupted absorbable sutures.

All animal procedures were performed in accordance with the National Institutes of Health Animal Care and Use Guidelines. All animal protocols were approved by the Animal Ethics Committee at the Beijing Institute of Geriatrics.

### Microarray analysis for miRNAs

Microarray analysis was performed by Kangcheng Bio-tech Inc. (Shanghai, China). To profile the expression of miRNAs, the miRNAs in the liver samples from 5 db/db mice and 5 control mice were analyzed by the miRCURY^TM^ LNA Array (v.14.0 Exiqon). Total RNA was harvested using TRIzol (Invitrogen) and an RNeasy mini kit (Qiagen) according to the manufacturer’s instructions. The samples were labeled using the miRCURY^TM^ Hy3^TM^/Hy5^TM^ Power labeling kit and hybridized on the miRCURY^TM^ LNA Array (v.14.0 Exiqon). Scanning was performed with an Axon GenePix 4000B microarray scanner (Molecular Devices, Downingtown, PA, USA). GenePix pro V6.0 (Molecular Devices) was used to read the raw intensity of the image. The intensity of the green signal was calculated after background subtraction, and four replicated spots of each probe on the same slide were averaged. The median normalization method was used to obtain “Normalized Data”: Normalized Data = (Foreground-Background)/median, where the median was the 50% quartile of the miRNA intensity, which was larger than 50 in all samples after background correction^25^.

### Cell culture

NCTC 1469 cells and HEP 1–6 cells were derived from mouse liver cells (American Type Culture Collection). NCTC 1469 cells were cultured in low glucose Dulbecco’s modified Eagle’s medium (Invitrogen) supplemented with 20% horse serum (Hyclone), 100 units/ml penicillin (Invitrogen), and 0.1 mg/ml streptomycin (Invitrogen) at 37 °C in a humidified atmosphere of 95% O_2_ and 5% CO_2._ HEP 1–6 cells were cultured in high Dulbecco’s modified Eagle’s medium (Invitrogen) supplemented with 10% fetal serum (Hyclone), 100 units/ml penicillin (Invitrogen), and 0.1 mg/ml streptomycin (Invitrogen) at 37 °C in a humidified atmosphere of 95% O_2_ and 5% CO_2._ The cells were treated with 10 nM insulin for 10 min before harvesting the protein sample.

### Transfection of miRNA mimics and inhibitor

The mimic and inhibitor of miR-19a were purchased from Genepharm (Shanghai, China). The miRNA mimic control and inhibitor control were used as negative controls. Hiperfect transfection reagent (Qiagen) was used for the transfection of miR-19a mimics and inhibitor, and 48 h after transfection, the expression of miR-19a was detected by real-time PCR.

### Luciferase target assay

Three binding sites of miR-19a on 3'-UTR of PTEN were analyzed by miRNA target prediction databases, including Miranda, TargetScan and PicTar. The fragment of PTEN 3'-UTR from 500 nt to 1600 nt was cloned and inserted into pmiRGLO vector (PTEN500). A luciferase reporter assay was used to assess whether miR-19a can directly bind to the 3'-UTR of PTEN. For the luciferase assay, the 3'-untranslated region (UTR) of PTEN, including the binding sites for miR-19a, was amplified from NCTC 1469 cells using the following primers (restriction sites are underlined):

PTEN-UTR-F-Sac I: TCGAGCTCGGGTTCACGTCCTACCCCTTT

PTEN-UTR-R-Xba l: GCTCTAGAGC TTCGTGCAGTGCTGTAAATTT

PCR was performed with genome DNA isolated from NCTC 1469 cells, and the PCR product was then digested with Sac I and Xba I (NEB). Then, the fragment was inserted into the pmiRGLO (Promega) luciferase reporter vector. The procedures of the PCR are described as follows: a hot start step at 95 °C for 10 min, followed by 40 cycles at 95 °C for 15 s, 55 °C for 45 s, and 72 °C for 30 s.

To conduct the luciferase reporter assay, 5000 cells per well in 100 μl of medium were seeded in 96-well plates. After incubation overnight, the cells were transfected with the modified firefly luciferase vector and miR-19a mimics with Effectene Reagent (Qiagen) according to the manufacturer’s instruction, and 48 h after transfection, the firefly and renilla luciferase activities were measured with the dual-luciferase reporter assay system (Promega). To control the transfection efficiency, firefly activity was normalized to renilla activity.

### RNA isolation and real-time PCR

Total RNA was harvested using TRIzol (Invitrogen). Enriched miRNA was isolated using a miRNA isolation kit (TakaRa). A stem-loop reverse transcription-polymerase chain reaction (RT-PCR) was also executed on the samples to detect and quantify mature miRNAs using stem-loop antisense primer mix and avian myeloblastosis virus transcriptase (TaKaRa).

The cDNA preparations were routinely tested by real-time PCR based on the SYBR Green I method, according to the manufacturer’s instructions (TaKaRa). The amplification and detection of specific products were performed according to the manufacturer’s protocol with the iQ5 system (BioRad). The U6 small nucleolar RNA was used as the housekeeping small RNA reference gene. The relative gene expression was normalized to U6 small nucleolar RNA. Each reaction was performed in triplicate, and analysis was performed by the 2^−△△CT^ method. The nucleotide primers used for reverse transcription were as follows (5'−3'): miR-19a, GTCGTATCCAGTGCAGGGTCCGAGGTATTCGCACTGGATACGACTCAGTT; U6, GTCGT ATCCAGTGCAGGGTCCGAGGTATTCGCACTGGATACGACAAAA ATATG. The nucleotide primers used for real-time PCR were as follows (5'-3'): miR-19a forward, GCGTGTGCAAATCTATGCAA; U6 forward, GCGCGTCGTGAAGCGTTC; universal reverse primer, GTGCAGG GTCCGAGGT.

### Western blot analysis

Cell lysates (15 μg of protein) were separated by 10% SDS-PAGE, transferred to PVDF membranes (Millipore), blocked with 8% nonfat dry milk, and probed with the antibodies at 4 °C overnight. The blots were incubated with HRP-conjugated anti-IgG, followed by detection with ECL (Millipore). The antibodies against AKT, phosphorylation of AKT (Ser^473^), PTEN, glycogen synthase kinase (GSK), and phosphorylation of GSK (Ser^9^) were purchased from Cell Signaling.

### Measurement of glycogen content

The glycogen levels were measured in the cells or liver tissues incubated for 3 h in the presence of 1 nM insulin (Usbio), using a glycogen assay kit (Biovision).

### Statistical analysis

All values are represented as the means ± S.E. of the indicated number of measurements. A one-way analysis of variance test was used to determine significance, with a value of p < 0.05 indicating statistical significance.

## Additional Information

**How to cite this article**: Dou, L. *et al*. MiR-19a regulates PTEN expression to mediate glycogen synthesis in hepatocytes. *Sci. Rep*. **5**, 11602; doi: 10.1038/srep11602 (2015).

## Supplementary Material

Supplementary Information
