# Supplementary material for: MiR-19a regulates PTEN expression to mediate glycogen synthesis in hepatocytes
Source: Sci Rep. 2015 Jun 26;5:11602. doi: 10.1038/srep11602 (PMC4481380; doi:10.1038/srep11602)
Supplement: Supplementary Information [file srep11602-s1.pdf]

Supplementary information for

MiR-19a regulates PTEN expression to mediate glycogen synthesis in hepatocytes

Lin Dou[1*], XiangyuMeng [2,3*], Xiaofang Sui[4], Shuyue Wang[2,3], Tao Shen[2], Xiuqing Huang[2], Jun Guo[2], Weiwei Fang[2], Yong Man[2], Jianzhong Xi[1#], Jian Li[2,3#]

1 Department of Biomedical Engineering, College of Engineering, Peking University, Beijing, China, 2Key Laboratory of Geriatrics, Beijing Institute of Geriatrics & Beijing Hospital, Ministry of Health, Beijing, China, 3 Peking University Fifth School of Clinical Medicine, Beijing, China, 4 First Affiliated Hospital of Jiamusi University, Jiamusi, China

[*]These authors contributed equally to this work.

[#] Correspondence and requests for materials should be addressed to J.L.(lijian@bjhmoh.cn); J.X.(jzxi@pku.edu.cn)

Supplementary Figure S2 is related to Figure. 2e, 2f, 2k and 2l

Supplementary Figure S3 is related to Figure. 3a, 2b, 3c and 3d

Supplementary Figure S4 is related to Figure. 4d, 4e, 4f and 4g

Supplementary Figure S5 is related to Figure. 5a, 5d and 5e

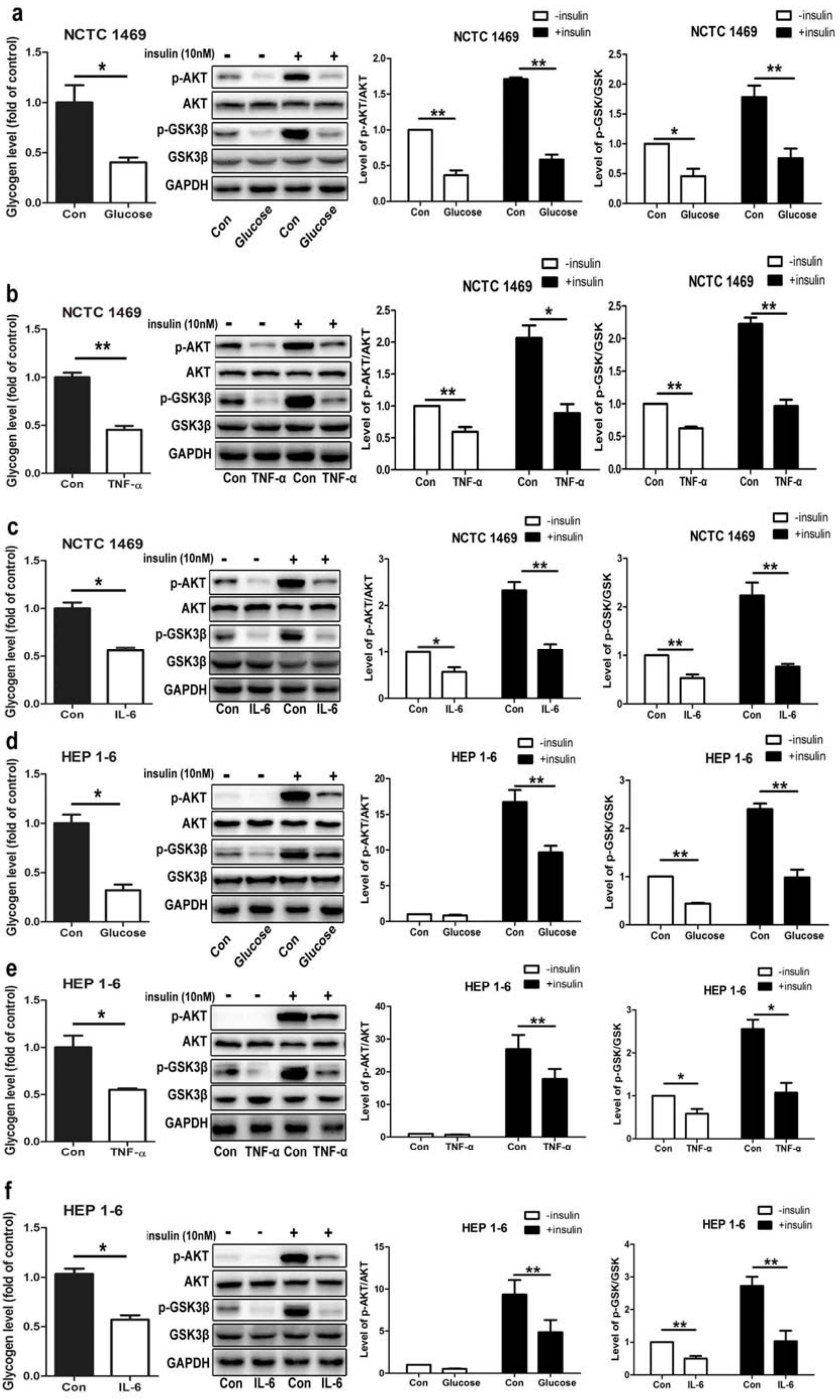

a
b
c
d
e
f
NCTC 1469
HEP 1-6
Glycogen level (fold of control)
Con
Glucose
TNF-α
IL-6
insulin (10nM)
p-AKT
AKT
p-GSK3β
GSK3β
GAPDH
Level of p-AKT/AKT
Level of p-GSK/GSK
-insulin
+insulin

Supplementary Figure S1. The activation of AKT/GSK pathway and synthesis of glycogen in hepatocytes were impaired in NCTC1469 cells and HEP 1-6 cells treated with high glucose, TNF-α and IL-6 respectively. Activation of the AKT/GSK pathway and synthesis of glycogen in the NCTC 1469 cells and HEP 1-6 cells treated with 33.3 mM glucose for 48 h (a and d), 10 ng/ml TNF-α for 24 h (b and e), 10 ng/ml IL-6 for 24 h (c and f), respectively.

A

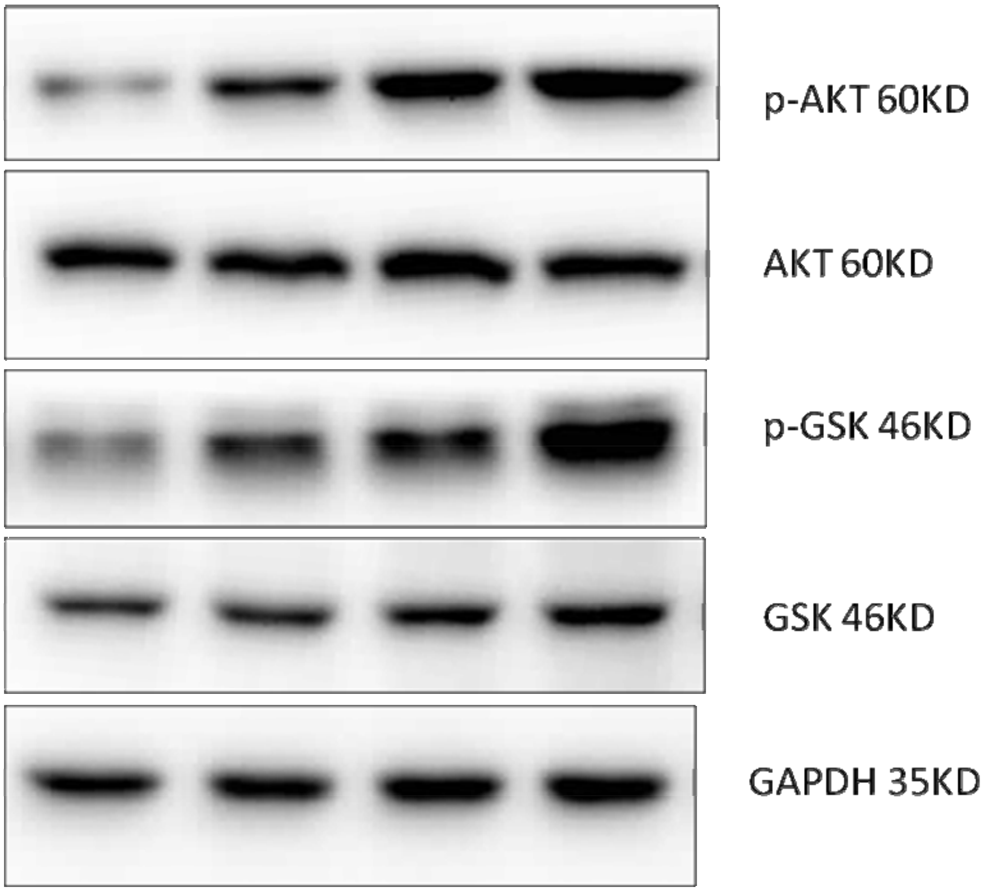

B

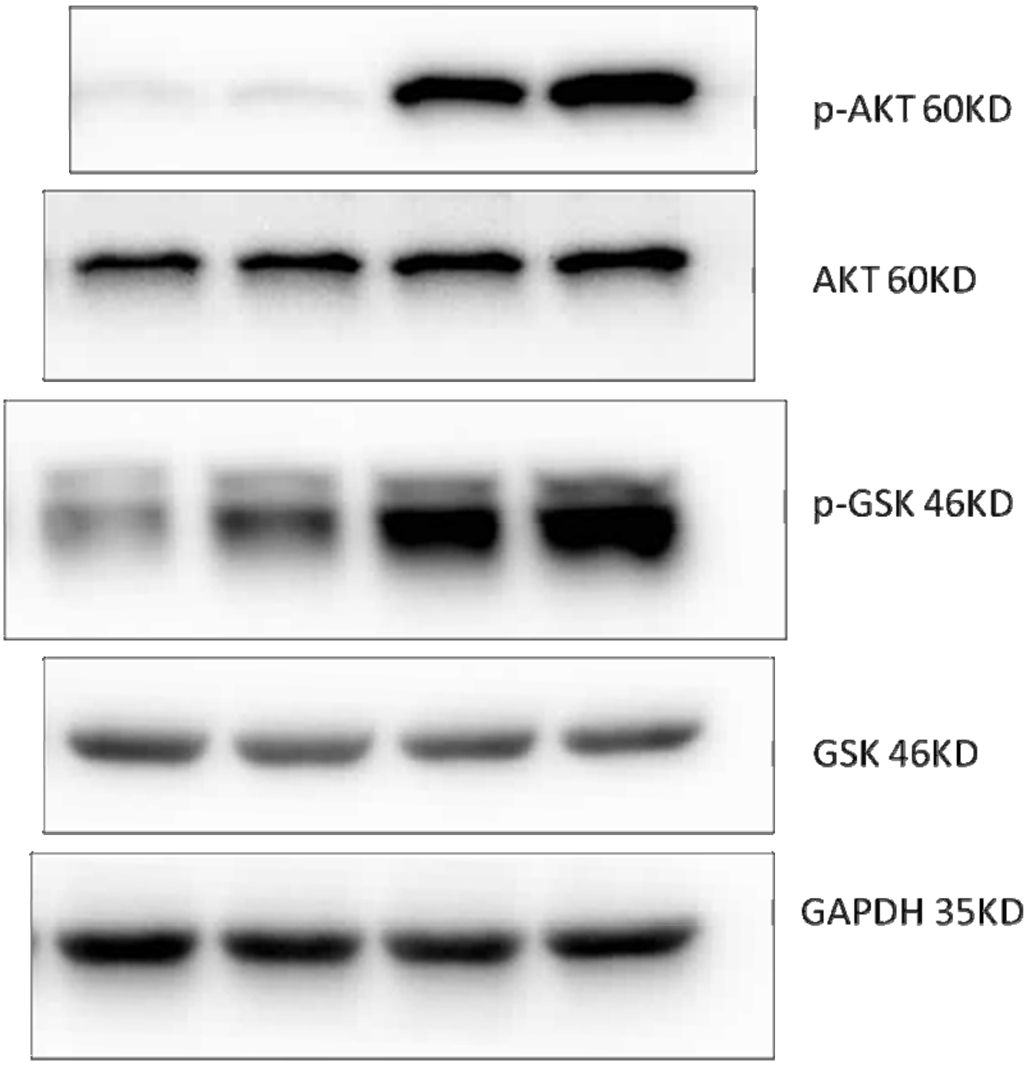

C

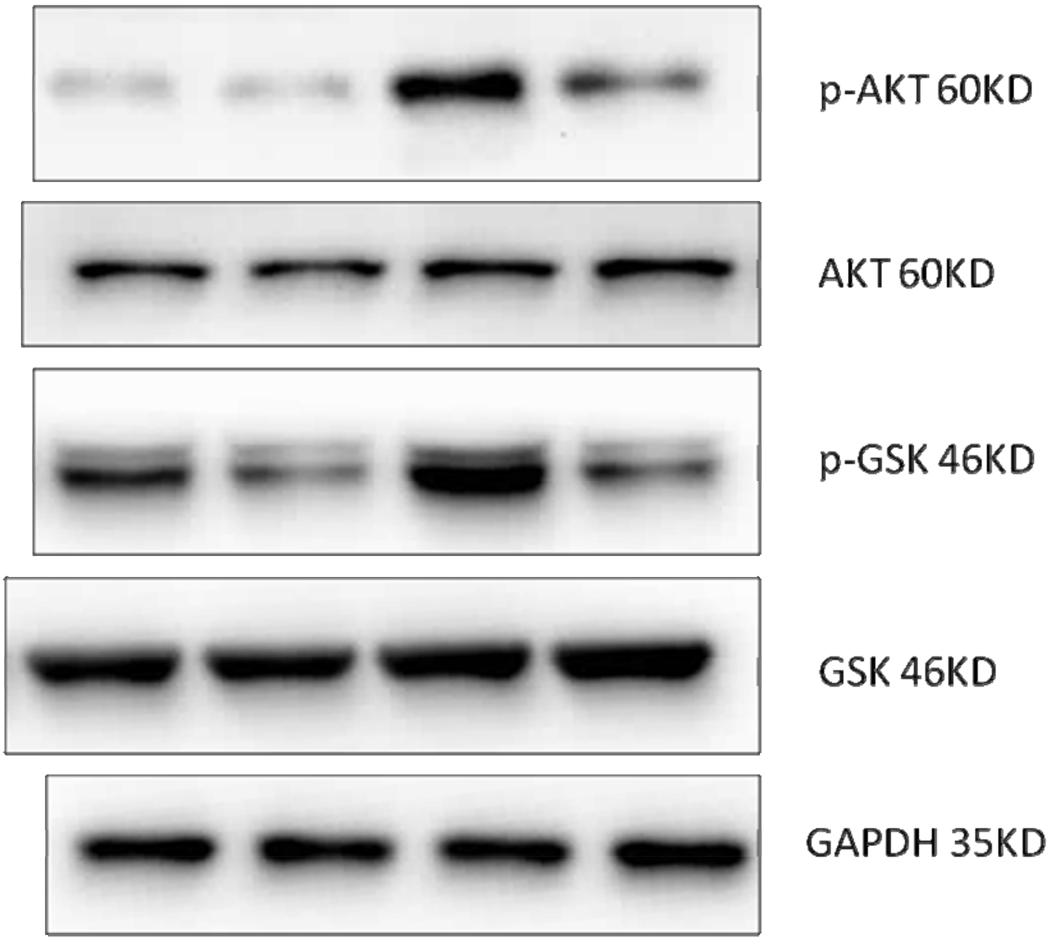

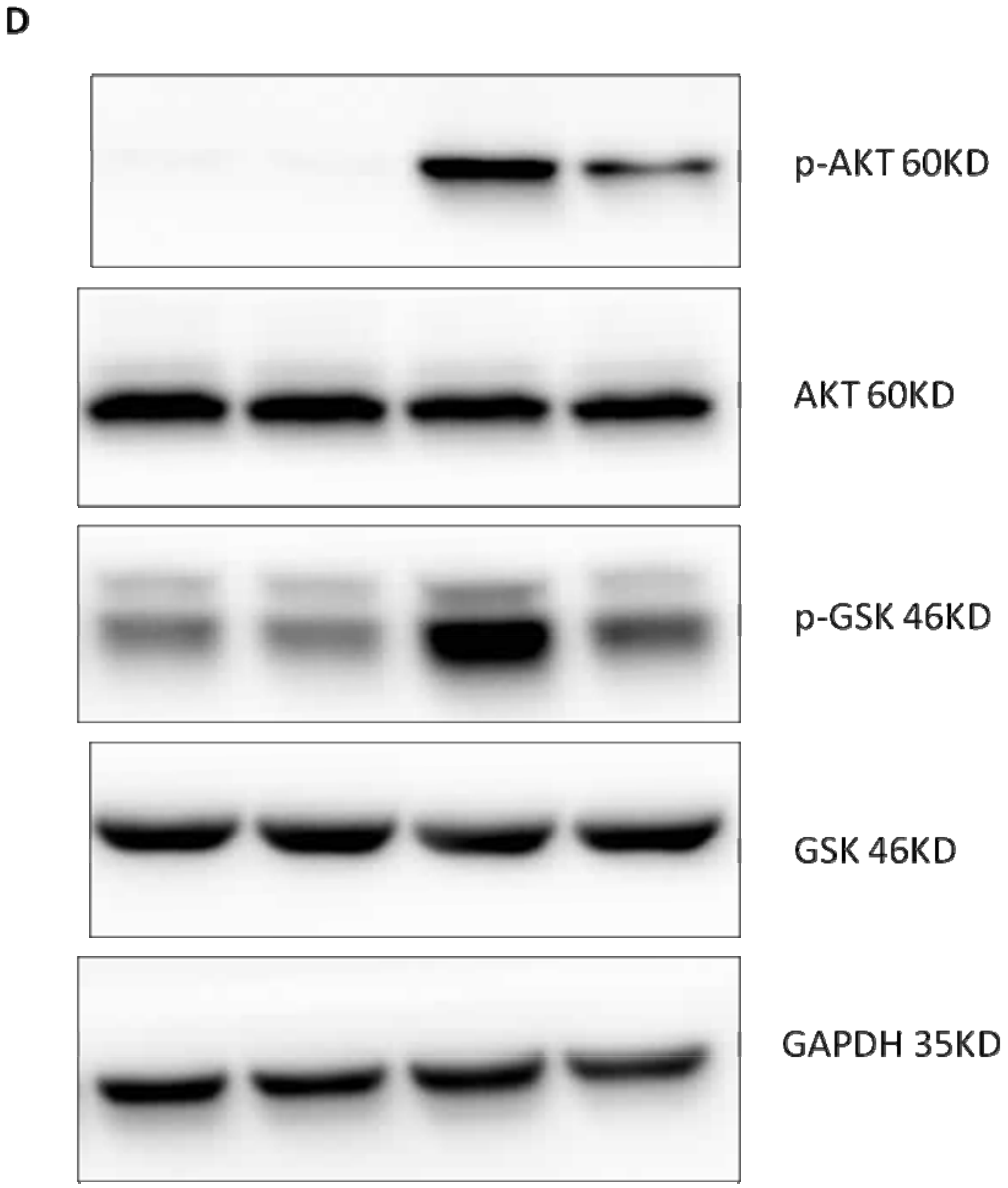

Supplementary Figure S2. Full-length blots of Figure. 2 to detect protein expressions of p-AKT, AKT, p-GSK, GSK, GAPDH in NCTC 1469 cells and HEP 1-6 cells transfected with miR-19a mimic and inhibitor.

A

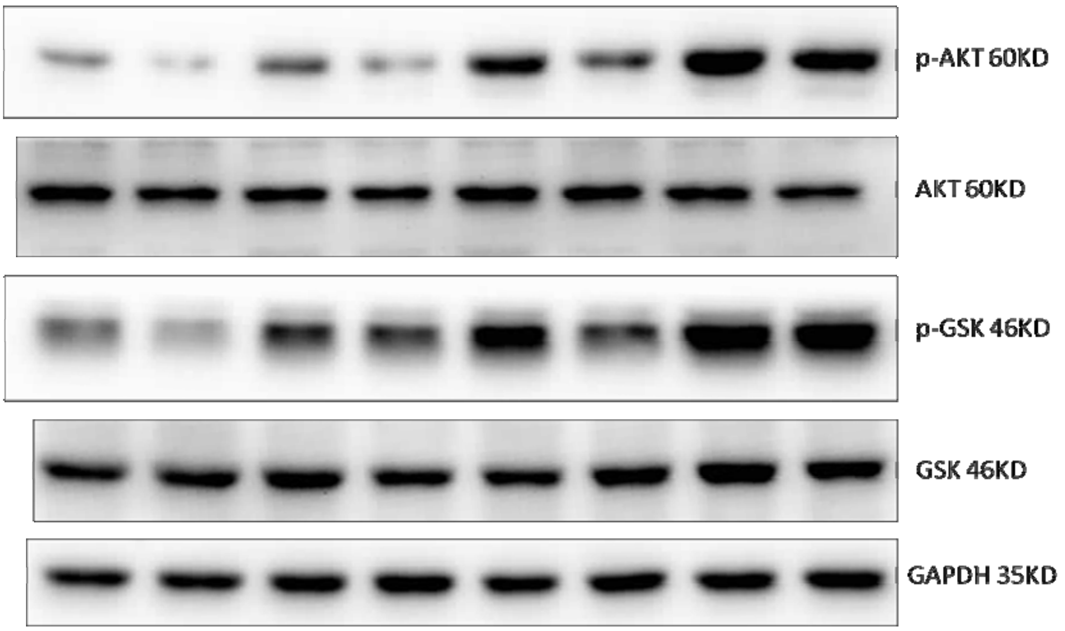
p-AKT 60KD
AKT 60KD
p-GSK 46KD
GSK 46KD
GAPDH 35KD

B

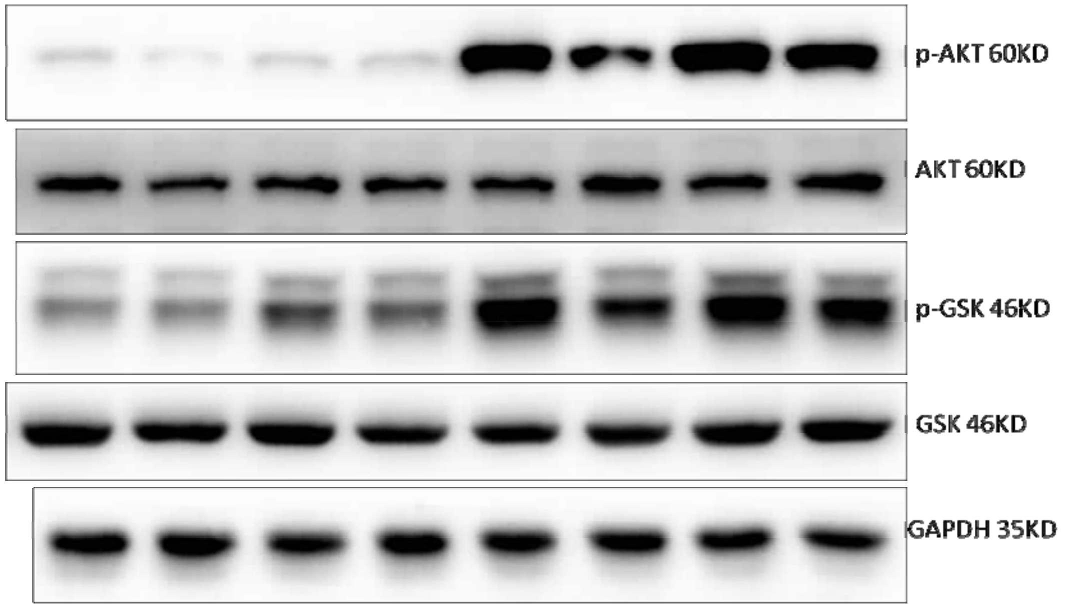
p-AKT 60KD
AKT 60KD
p-GSK 46KD
GSK 46KD
GAPDH 35KD

C

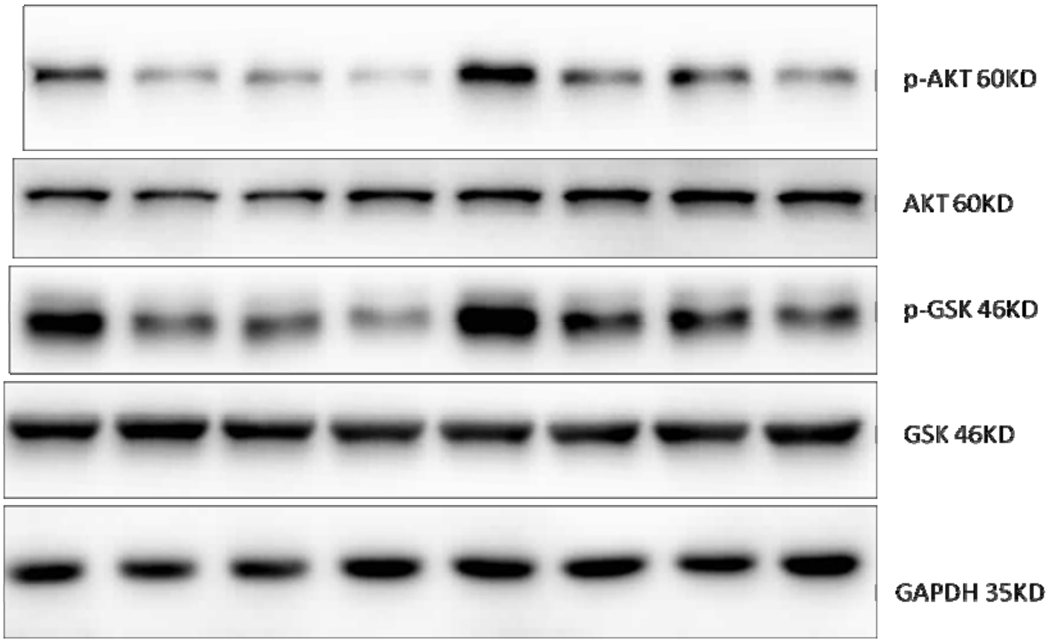

D

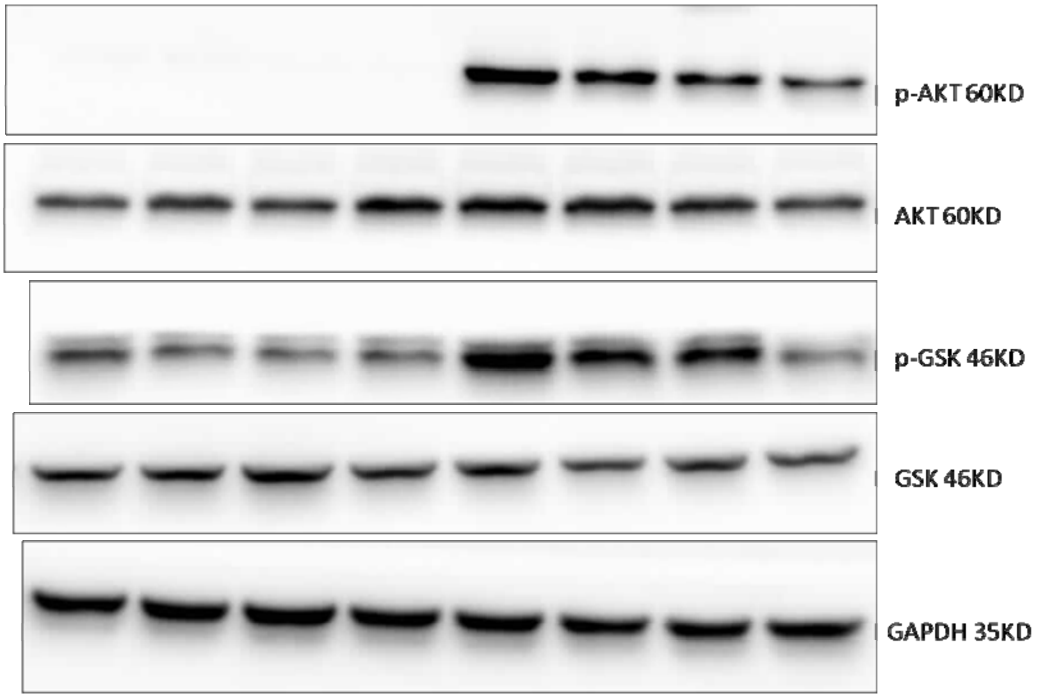

Supplementary Figure S3. Full-length blots of Figure. 3 to detect protein expressions of p-AKT, AKT, p-GSK, GSK, GAPDH in NCTC 1469 cells and HEP 1-6 cells treated by 10 ng/ml IL-6 for 24 h followed by transfection with miR-19a mimic or inhibitor for 48 h.

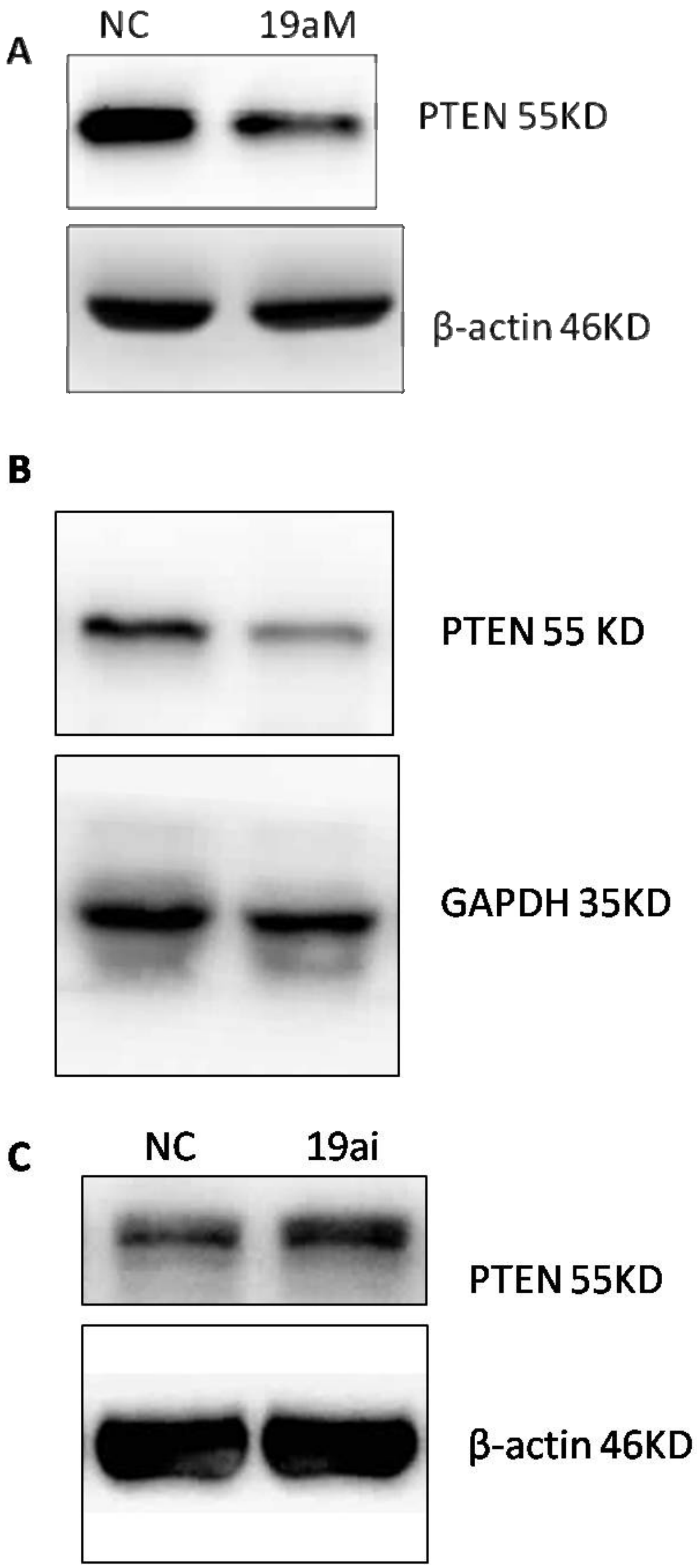
A
NC
19aM
PTEN 55KD
β-actin 46KD
B
PTEN 55 KD
GAPDH 35KD
C
NC
19ai
PTEN 55KD
β-actin 46KD

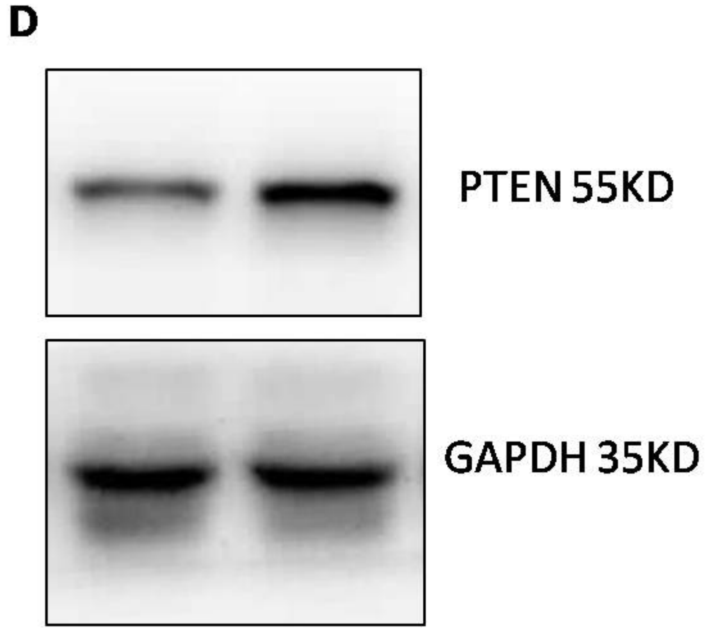

Supplementary Figure S4. Full-length blots of Figure. 4 to detect protein expressions of PTEN, β-actin and GAPDH in NCTC 1469 cells and HEP 1-6 cells transfected with miR-19a mimic or inhibitor for 48 h.

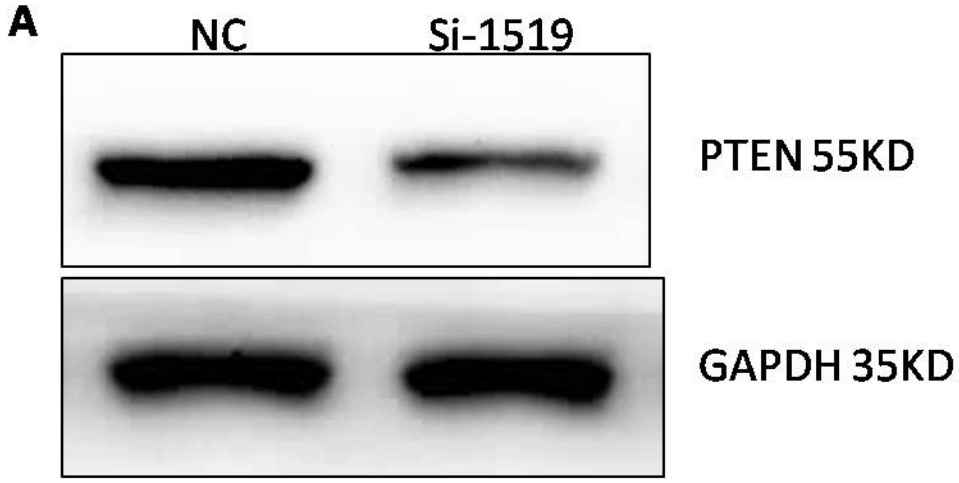

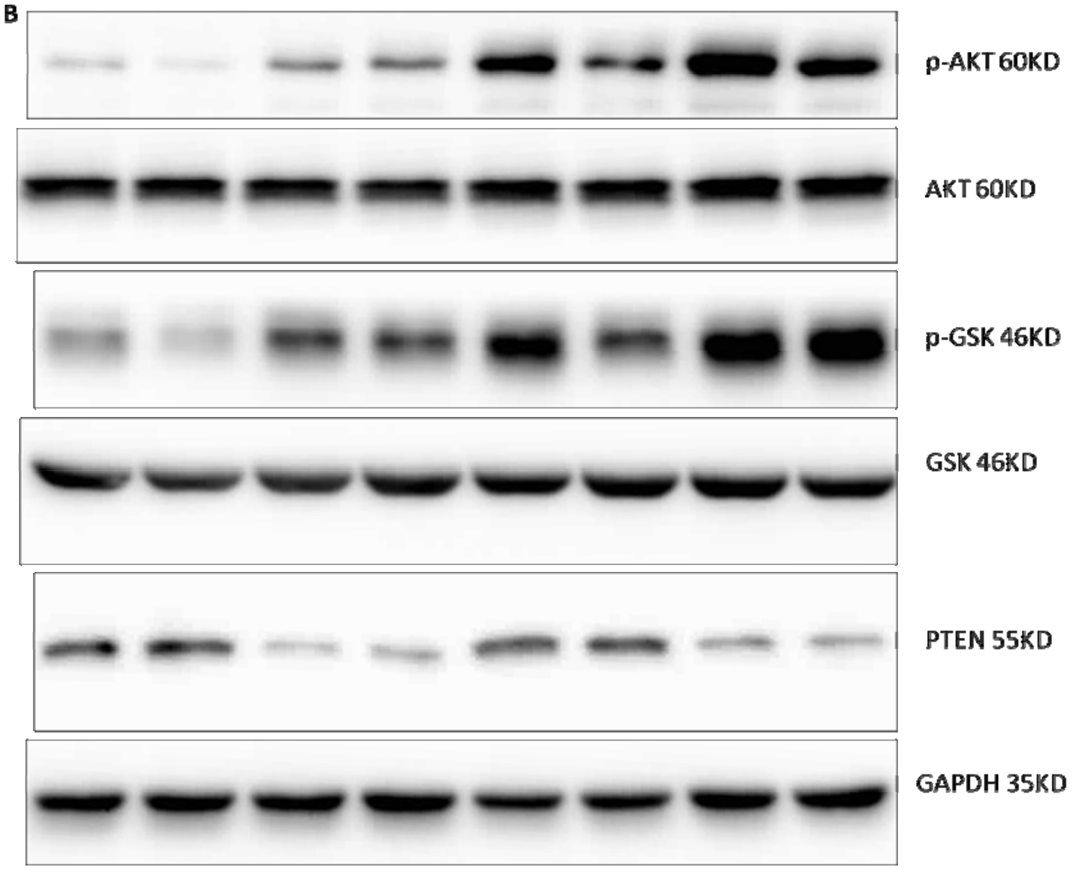
B
p-AKT 60KD
AKT 60KD
p-GSK 46KD
GSK 46KD
PTEN 55KD
GAPDH 35KD

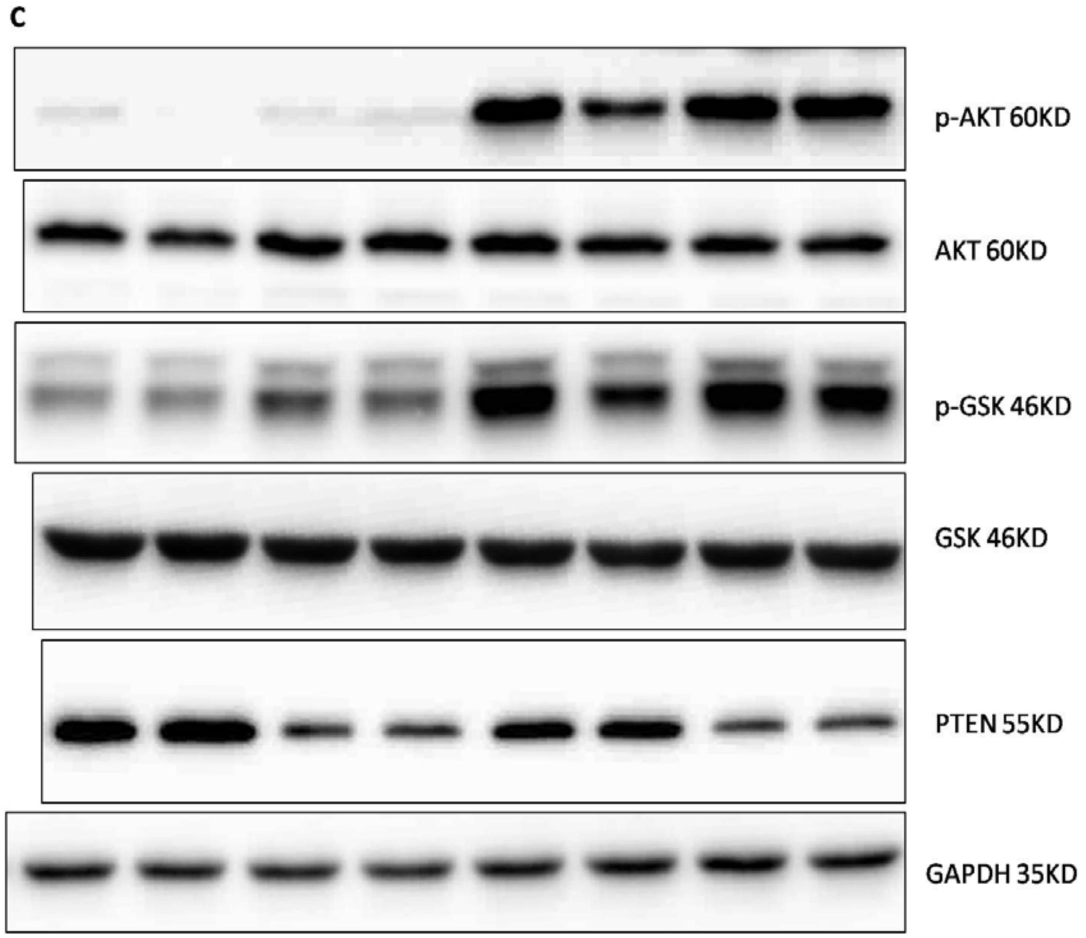

Supplementary Figure S5. (A) Full-length blots of Figure. 5a to detect protein expressions of PTEN and β-actin in NCTC 1469 cells transfected with PTEN-specific-siRNA for 48 h. (B and C) Full-length blots of Figure. 5d ang 5e to detect protein expressions of p-AKT, AKT, p-GSK, GSK, GAPDH in NCTC 1469 cells and HEP 1-6 cells treated with 10 ng/ml IL-6 for 24 h followed by transfection with PTEN-specific-siRNA for 48 h.

Supplementary Table 1. The results of microRNA microarray analysis

Name	Change	Foreground		Foreground-background		Normalized	
	(D/C)	C	D	C	D	C	D
mmu-miR-665	1.400566406	146.5	182	79.5	112	0.311765	0.4366472
mmu-miR-207	1.549422782	2786.5	4314	2720.5	4240	10.66863	16.530214
mmu-miR-709	1.472661734	7498	11076.5	7429.5	11005.5	29.13529	42.906433
mmu-miR-200c	1.369990016	108.5	123	41	56.5	0.160784	0.2202729
mmu-miR-720	1.729466953	13968.5	24253	13900	24181	54.5098	94.272904
mmu-miR-1897-5p	1.383657163	422	559	353.5	492	1.386275	1.9181287
mmu-miR-711	1.325536062	133.5	155.5	64.5	86	0.252941	0.3352827
mmu-miR-434-3p	1.510036352	159	211.5	92.5	140.5	0.362745	0.5477583
mmu-miR-677	1.701988304	196	283.5	125	214	0.490196	0.834308
mmu-miR-463	2.861952862	102	170.5	33	95	0.129412	0.3703704
mmu-miR-2134	1.554570371	297	434	231.5	362	0.907843	1.411306
mmu-miR-291b-3p	1.883231113	131	192.5	61.5	116.5	0.241176	0.454191
mmu-miR-2140	1.328130066	837	1096	766.5	1024	3.005882	3.9922027
mmu-miR-183*	1.623503202	249.5	362.5	178.5	291.5	0.7	1.1364522
mmu-miR-224	1.695156695	108	143.5	39	66.5	0.152941	0.2592593
mmu-miR-291b-5p	1.456345542	127	161.5	57	83.5	0.223529	0.3255361
mmu-miR-685	1.338281601	122	148	52	70	0.203922	0.2729045
mmu-miR-668	1.461203344	289	399	223.5	328.5	0.876471	1.2807018
mmu-miR-2132	1.479188166	2377.5	3504.5	2312	3440	9.066667	13.411306
mmu-miR-375	1.767663206	268.5	424.5	196	348.5	0.768627	1.3586745
mmu-miR-351	1.7620488	283	457.5	217.5	385.5	0.852941	1.502924
mcmv-miR-m108-1	4.931773879	97	196.5	25.5	126.5	0.1	0.4931774
mmu-miR-1959	1.596491228	239.5	349	170	273	0.666667	1.0643275
mmu-miR-190	1.500606863	118	151	53	80	0.207843	0.3118908
mmu-miR-1274a	1.52714958	1090	1639.5	1024	1573	4.015686	6.1325536
mmu-miR-1937c	1.4260009	287.5	387	221	317	0.866667	1.2358674
mghv-miR-M1-8	1.71824381	488.5	797	421.5	728.5	1.652941	2.8401559
mmu-miR-705	1.707902234	105	140.5	39	67	0.152941	0.2612086
mmu-miR-2137	1.440100251	240.5	321	175	253.5	0.686275	0.9883041
mmu-miR-290-5p	1.848057644	352	596.5	280	520.5	1.098039	2.0292398
mmu-miR-615-3p	1.886165185	141	211	73	138.5	0.286275	0.539961
mmu-miR-142-3p	0.538225395	3625.5	1995.5	3557.5	1926	13.95098	7.5087719
mmu-miR-574-5p	0.730994152	150	136	85	62.5	0.333333	0.2436647
mmu-miR-181a	0.171261487	187	93	119	20.5	0.466667	0.079922
mmu-miR-130a	0.480917864	2404	1197	2338	1131	9.168627	4.4093567
mmu-miR-10a	0.49129607	236.5	153	172	85	0.67451	0.331384

mmu-miR-19b	0.655781019	1227	836.5	1162	766.5	4.556863	2.9883041
mmu-miR-151-5p	0.644931498	248.5	190.5	176.5	114.5	0.692157	0.4463938
mmu-miR-470	0.703180716	130.5	119.5	61.5	43.5	0.241176	0.1695906
mmu-miR-466f-3p	0.679884974	312.5	244	242	165.5	0.94902	0.6452242
mmu-miR-24	0.680479415	1714	1203.5	1646.5	1127	6.456863	4.3937622
mmu-miR-713	0.761222702	278	230	207	158.5	0.811765	0.6179337
mmu-miR-669o	0.673633081	161.5	132.5	91.5	62	0.358824	0.2417154
mmu-let-7d	0.744819984	693	543.5	626	469	2.454902	1.82846
mmu-miR-29a*	0.346349745	148.5	102	77.5	27	0.303922	0.1052632
mmu-miR-706	0.726163234	190.5	158	115	84	0.45098	0.3274854
mmu-miR-669e	0.559210526	125	112	56	31.5	0.219608	0.122807
mmu-miR-148a	0.691179755	1999	1420	1924.5	1338	7.547059	5.2163743
mmu-miR-19a	0.621991142	362	258	288.5	180.5	1.131373	0.7037037
mmu-miR-24-2*	0.205403315	131	92.5	60.5	12.5	0.237255	0.0487329
mmu-miR-32	0.72028096	437.5	335.5	363	263	1.423529	1.0253411
mmu-miR-125b-5p	0.546882579	822	486	753.5	414.5	2.954902	1.6159844
mmu-miR-140	0.765986003	128.5	124.5	61	47	0.239216	0.1832359
mmu-miR-142-5p	0.471928838	658.5	356.5	593	281.5	2.32549	1.0974659
mmu-miR-27b	0.684797803	778	563.5	707	487	2.772549	1.8986355
mmu-miR-200b	0.476409843	298	179.5	228.5	109.5	0.896078	0.4269006
mmu-miR-125a-5p	0.598567184	494.5	325	431	259.5	1.690196	1.0116959
mmu-miR-101b	0.578569149	4718	2775	4654	2708.5	18.25098	10.559454
mmu-miR-374	0.650466072	281.5	207.5	215.5	141	0.845098	0.5497076
mmu-miR-200a	0.105150697	168.5	77	104	11	0.407843	0.042885
mmu-miR-15a	0.561135664	1178.5	706	1113.5	628.5	4.366667	2.4502924
mmu-miR-195	0.438528963	281.5	171.5	216.5	95.5	0.84902	0.3723197
mmu-miR-101a	0.701126412	6817	4832	6751.5	4761.5	26.47647	18.563353
mmu-miR-33	0.618803868	1066.5	696	1002.5	624	3.931373	2.4327485
mmu-miR-139-5p	0.670753188	193	157.5	124.5	84	0.488235	0.3274854
mmu-miR-693-5p	0.673532245	287.5	213	215.5	146	0.845098	0.5692008
mmu-miR-466a-5p	0.727745289	149.5	124	76.5	56	0.3	0.2183236
mmu-miR-140*	0.481845282	250.5	166.5	179.5	87	0.703922	0.3391813
mmu-miR-126-3p	0.635417719	3713.5	2408	3647	2331	14.30196	9.0877193
mmu-miR-148b	0.733430799	270.5	227.5	204	150.5	0.8	0.5867446
mmu-miR-669f	0.739909884	223.5	180.5	152.5	113.5	0.598039	0.4424951
mmu-miR-331-3p	0.64205653	167	133	96	62	0.376471	0.2417154
mmu-miR-1903	0.711454782	175	149.5	105.5	75.5	0.413725	0.294347
mmu-miR-2135	0.416354276	361	198.5	292.5	122.5	1.147059	0.4775828
mmu-miR-26b	0.581066352	2715.5	1623	2642.5	1544.5	10.36275	6.0214425
mmu-miR-106b	0.665169365	690.5	485.5	621	415.5	2.435294	1.619883
mmu-miR-26a	0.567637936	5128	2960.5	5061.5	2890	19.84902	11.267057
mmu-let-7b	0.754854756	2395.5	1837.5	2326.5	1766.5	9.123529	6.8869396
mmu-miR-23a	0.436572737	486	255.5	419	184	1.643137	0.7173489
mmu-miR-325	0.74287079	525.5	410	453	338.5	1.776471	1.3196881

mmu-miR-466i	0.454612242	170.5	117	99.5	45.5	0.390196	0.1773879
mmu-miR-27a	0.595798439	213.5	163	143.5	86	0.562745	0.3352827
mmu-miR-99a	0.741095162	149	132.5	82.5	61.5	0.323529	0.2397661
mmu-let-7g	0.66421197	2132	1458.5	2065.5	1380	8.1	5.380117
mmu-miR-199a-5p	0.448539981	215	139	148.5	67	0.582353	0.2612086
mmu-miR-146b	0.136809915	175	97	109	15	0.427451	0.0584795
mmu-let-7i	0.703071493	289	227	222	157	0.870588	0.6120858
mmu-miR-223	0.58240515	251	181	183.5	107.5	0.719608	0.4191033
mmu-miR-16	0.570344455	2489.5	1461	2422	1389.5	9.498039	5.417154
mmu-miR-22*	0.661760785	231	188	164.5	109.5	0.645098	0.4269006
mmu-miR-17	0.686212025	329.5	258	261.5	180.5	1.02549	0.7037037
mmu-let-7a	0.75168127	2339.5	1795.5	2273.5	1719	8.915686	6.7017544
mmu-let-7c	0.675192966	1124	785	1053.5	715.5	4.131373	2.7894737
mmu-miR-423-5p	0.672871202	148.5	126	82	55.5	0.321569	0.2163743
mmu-miR-338-3p	0.275606508	116	80.5	50.5	14	0.198039	0.0545809
mmu-miR-29a	0.69683204	8016	5640.5	7948	5571	31.16863	21.719298
mmu-miR-143	0.474304485	1276	649.5	1211.5	578	4.75098	2.2534113
mmu-miR-652	0.570175439	168.5	130	102	58.5	0.4	0.2280702
mmu-miR-362-3p	0.379585327	123.5	91	55	21	0.215686	0.0818713
mmu-miR-1839-3p	0.757899184	330	272	263	200.5	1.031373	0.7816764
mmu-miR-30c	0.726133294	2807	2082	2743	2003.5	10.75686	7.8109162
mmu-miR-339-5p	0.721943748	194.5	169	126	91.5	0.494118	0.3567251
mmu-miR-126-5p	0.661197491	1019.5	710	949.5	631.5	3.723529	2.4619883
mmu-miR-191	0.667216139	367	275.5	298	200	1.168627	0.7797271
mmu-miR-93	0.700976388	296	236.5	225.5	159	0.884314	0.619883
mmu-miR-144	0.763726873	1403	1098	1331	1022.5	5.219608	3.9863548
mmu-miR-669l	0.745614035	145	129	78	58.5	0.305882	0.2280702
mmu-miR-20a	0.649281472	332.5	239	258	168.5	1.011765	0.6569201
mmu-miR-301a	0.355705778	120	92	54.5	19.5	0.213725	0.0760234
mmu-miR-199a-3p	0.483641536	159	116	92.5	45	0.362745	0.1754386
mmu-miR-145	0.641919368	221.5	167.5	151	97.5	0.592157	0.380117
mmu-miR-185	0.550813972	142.5	112.5	74	41	0.290196	0.1598441
mmu-miR-465b-5p	0.64369557	205.5	158.5	139	90	0.545098	0.3508772
mmu-miR-214	0.488103532	204	146	138.5	68	0.543137	0.2651072
mmu-miR-466d-5p	0.644210526	130.5	117	62.5	40.5	0.245098	0.1578947
mmu-miR-678	0.54172357	153	123	83.5	45.5	0.327451	0.1773879
mmu-miR-146a	0.206104693	195.5	100.5	123	25.5	0.482353	0.0994152
mmu-miR-23b	0.502999313	1621.5	859	1552.5	785.5	6.088235	3.0623782
mmu-miR-30b	0.739335958	2760	2077.5	2692	2002	10.55686	7.8050682

D: db/dbmouse   C: wild type mouse
